# Wastewater Surveillance for SARS-CoV-2 in Rural Kentucky, 2021–2023

**DOI:** 10.3390/v18030282

**Published:** 2026-02-26

**Authors:** James W. Keck, Reuben Adatorwovor, Ann Noble, Savannah Tucker, William D. Strike, Soroosh Torabi, Mohammad Dehghan Banadaki, Blazan Mijatovic, Steven K. Roggenkamp, Donna L. McNeil, Lindell E. Ormsbee, Scott M. Berry

**Affiliations:** 1 Department of Family and Community Medicine, University of Kentucky, Lexington, KY 40506, USA; 2 Department of Biostatistics, University of Kentucky, Lexington, KY 40506, USA; 3 Department of Mechanical and Aerospace Engineering, University of Kentucky, Lexington, KY 40506, USA; 4 Department of Biomedical Engineering, University of Kentucky, Lexington, KY 40506, USA; 5 Institute for Biomedical Informatics, University of Kentucky, Lexington, KY 40506, USA; 6 Kentucky Water Research Institute, University of Kentucky, Lexington, KY 40506, USA; 7 Department of Civil Engineering, University of Kentucky, Lexington, KY 40506, USA; lindell.ormsbee@uky.edu

**Keywords:** wastewater surveillance, SARS-CoV-2, COVID-19, rural, epidemiologic model

## Abstract

Wastewater testing for SARS-CoV-2 provided useful public health information during the COVID-19 pandemic yet was underutilized in rural communities. We addressed this gap by implementing wastewater surveillance and assessing its performance in 10 communities in Eastern Kentucky. We collected wastewater samples 1–2 times weekly at 10 wastewater treatment plants (WWTPs) from May 2021 until April 2023 and measured SARS-CoV-2 RNA concentrations using polymerase chain reaction testing. We calculated time-lagged correlations between wastewater concentrations and county-level reported COVID-19 cases by site. We developed a generalized linear model to estimate COVID-19 incidence from wastewater SARS-CoV-2 concentrations. The 10 participating WWTPs served 2430 to 35,575 customers, and 90% were in rural counties. We cumulatively analyzed 818 wastewater samples. Correlations between wastewater SARS-CoV-2 concentrations and COVID-19 cases were significant at seven of the WWTPs and were strongest during the Delta variant period. The incidence density model predicted more COVID-19 cases during the latter study period (May 2022–April 2023) than were officially reported. Wastewater surveillance data in these rural communities corroborated clinical case data and may have more accurately described community disease levels later in the pandemic.

## 1. Introduction

The COVID-19 pandemic elevated wastewater-based disease surveillance (WBDS) in the United States as a tool to monitor SARS-CoV-2 virus levels in communities. Early adopters of WBDS demonstrated its sensitivity in detecting the virus and its correlation with COVID-19 cases in a community [1,2,3]. Recognizing the public health potential of WBDS, the Centers for Disease Control and Prevention (CDC) established the National Wastewater Surveillance System (NWSS) in September 2020 to coordinate wastewater surveillance activities across jurisdictions [4]. By 2021, 293 jurisdictions had submitted data to NWSS, and this grew to 671 jurisdictions by 2022 [5]. However, in 2021 only 13 states had participating jurisdictions and fewer than 5% were located in the most rural counties [5]. A similar disparity in the distribution of WBDS was noted in California; as of 2021 most WBDS sites were in urban coastal areas [6].

The limited early implementation of WBDS in rural communities may have been due to limited resources, laboratory capacity, personnel, competing pandemic priorities, and WBDS implementation decisions made by centralized state public health systems [7,8,9,10]. Unfortunately, many of these same factors also left rural communities vulnerable to COVID-19 with less access to clinical testing and healthcare and limited information about the local epidemiology of the pandemic to inform public health responses. Implementing WBDS in rural settings could offer an efficient means to monitor local SARS-CoV-2 trends and inform public health officials and community members [11].

In May 2021 we began WBDS for SARS-CoV-2 in several communities in rural Eastern Kentucky (Appalachia), and by the end of 2021, we were collecting weekly wastewater samples from 10 wastewater treatment plants across nine counties. Eight of these counties were nonmetropolitan by Rural-Urban Continuum Code designation and three were designated as “most rural.” This manuscript describes 2 years of SARS-CoV-2 wastewater data from these counties and compares the wastewater SARS-CoV-2 signal with publicly reported COVID-19 case data from the same counties.

## 2. Materials and Methods

### 2.1. Wastewater Sampling

In March of 2021, we began contacting the managers of wastewater treatment plants (WWTPs) in Eastern Kentucky to gauge interest in participating in WBDS for SARS-CoV-2. The partner utilities were selected based on previous relationships developed through a water and wastewater utility support program administered by the Kentucky Water Research Institute. We met with interested WWTPs, and enrolled plants signed a data use agreement. WWTP staff collected 24-h composite influent wastewater samples 1 or 2 times per week. One WWTP (site I) switched to grab samples to time sample collection with the influx of wastewater from holding tanks that stored wastewater until it reached a threshold volume, which triggered pumping the stored wastewater to the WWTP. Wastewater samples were refrigerated onsite and transported on wet ice by a courier to the University of Kentucky for processing. Study team staff entered wastewater sample metadata, such as collection time, sample temperature, and the plant’s daily flow rate, into a custom REDCap database. WWTP staff received no compensation for their time.

### 2.2. Wastewater Analysis

We typically processed wastewater samples within 20 h of sample collection. We analyzed eight 250 μL replicates from each sample to mitigate the heterogeneity of biological analytes in wastewater. Nucleic acids were extracted using the exclusion-based sample preparation (ESP) method. Strike et al. provide a detailed description of this method [12]. Briefly, sample replicates were lysed with a chaotropic buffer and combined with paramagnetic particles (PMPs; SeraSil-Mag Cytiva #29357369 and #29357374). These mixtures were vortexed and incubated at 50 °C for 20 min, tumbled at ambient temperatures for another 20 min, and pipet transferred onto ESP devices (Extractman, Gilson Inc., Middleton, WI, USA). These devices use magnets to efficiently manipulate each replicate’s PMPs within and between different wash buffers, as previously described. Purified nucleic acids bound to PMPs were incubated at 70 °C for 20 min to aid the final elution in 100 μL water. We created extraction control samples by diluting heat-inactivated SARS-CoV-2 (procured from BEI Resources) in wastewater that previously tested negative for SARS-CoV-2 RNA. These controls were stored at −80 °C and regularly extracted in parallel to measure assay efficiency over time.

SARS-CoV-2 RNA was quantified using the CDC’s N1 assay [13] provided by ThermoFisher (Waltham, MA, USA) in a 20× stock solution. We combined 10 μL of extracted nucleic acid with 5 μL of TaqMan™ Fast Virus 1-Step Master Mix (Applied Biosystems Cat# 4444434, Waltham, MA, USA), 4 μL water, and 1 μL of the 20× primers and probe mix. Each qPCR plate contained a positive control (spiked in SARS-CoV-2 genomic RNA from BEI resources) and negative control (no template RNA added). Plates were run using the following thermocycling program: 50 °C for 5 min, 95 °C for 20 s, and 45–50 cycles alternating between 95 °C for 15 s and 60 °C for 60 s. Data analysis utilized the Roche LightCycler 480 II software. Specifically, the 2nd Derivative Maximum Algorithm was used to determine quantification cycle (Cq) values for each well after visual confirmation with the amplification curve. Each Cq value was transformed into copies per milliliter using our static reference standard curve, which was created using serial dilutions of a BEI SARS-CoV-2 quantification control (r^2^ = 0.985) run in triplicate.

### 2.3. Data Management and Sharing

We constructed a data management pipeline that linked qPCR text file results with a database containing sample metadata. From this database, we created custom reports and data exports for CDC NWSS. We also created site-specific WBDS dashboards using Microsoft PowerBI that depicted the site’s wastewater SARS-CoV-2 concentrations over time in conjunction with county-level COVID-19 clinical case counts (as taken from the New York Times COVID-19 tracker GitHub archive [14]). Microsoft Power Automate alerted collaborators of new wastewater results via an e-mail notification.

### 2.4. Statistical Analysis

#### 2.4.1. Clinical Case Data

We extracted county-level SARS-CoV-2 positive clinical test data from the New York Times COVID-19 Tracker website [14]. The county-level COVID-19 data was paired with the corresponding WWTP wastewater data by date. Some wastewater test result dates lacked corresponding clinical case counts (*n* = 13). Additionally, there were 29 instances when reported cumulative case counts within a county decreased from the previous date (due to revision of case counts), resulting in negative values. We censored these data points and used the last observation carried forward method to impute both missing and censored values because it maintains continuity of each subject’s measurement over time, is straightforward to apply, and is widely accepted for use in longitudinal analyses. Daily clinical case data was normalized based on the estimated population served by the WWTP. We calculated seven-day moving averages of clinical cases to smooth day-of-the-week testing patterns. Considering that infected individuals can shed the virus before and after clinical testing results, as well as after recovering from infection, we conducted time-shifted (forward and backward by 7 days) correlational analyses. For instance, a one-day prior shift moves the date of wastewater collection one day prior to clinical test results from the same date, and a one-day post shift is defined similarly.

#### 2.4.2. SARS-CoV-2 Variant Circulation

Additionally, we generated analytic sample frames aligned with the circulation of COVID-19 variants. Wastewater samples collected from 23 May 2021 to 31 December 2021, were categorized as Delta variant-predominant and samples collected from 1 January 2022 to 31 May 2022, were categorized as Omicron BA.1 variant-predominant. Samples collected from 1 June 2022 to 30 October 2022, were categorized as Omicron BA.4/5 variant-predominant, while samples collected from 1 November 2022 to 17 April 2023, were categorized as Omicron BQ.1 variant-predominant. We applied these date ranges based on published US variant surveillance data [15,16].

#### 2.4.3. Wastewater Data

Each wastewater observation represents the arithmetic average of 8 replicates (or the number of analyzed replicates with valid PCR results) reported as genome copies per milliliter (gc/mL). We summarized measured wastewater data by WWTP using means, standard deviations, and graphical representations. To address outlier values, we identified replicates with Cq values in the top 3% for that site and replaced those Cq values with a ceiling value corresponding to the 97th percentile of that location’s wastewater SARS-CoV-2 distribution. We did this to preserve the observation sample size while mitigating the effect of extreme outlier values on data analysis. We used the dataset with ceiling values for subsequent analyses, including a descriptive analysis of wastewater data by SARS-CoV-2 variant.

We applied a nonparametric correlation method to ascertain the association between clinical cases and wastewater SARS-CoV-2 RNA levels. Specifically, we estimated Kendall’s Tau correlation coefficient to assess the relationship between the number of positive clinical cases and levels of wastewater SARS-CoV-2 RNA. We report Kendall’s Tau correlation coefficients because it provides a conservative and robust measure of association in the presence of tied ranks and non-normal data found in our clinical and wastewater datasets.

#### 2.4.4. Wastewater Prediction Model

To develop a COVID-19 case prediction model based on wastewater surveillance data, we first split the data into two datasets from the earlier pandemic period (May 2021–April 2022) and the later pandemic period (May 2022–April 2023). We selected 30 April 2022, as the partition date for several reasons. First, it corresponded to a natural break between waves of reported COVID-19 cases. Second, it served to partition the data into two similarly sized data sets. And third, there was an increasing trend of home COVID-19 testing during 2022 [17], which resulted in fewer positive COVID-19 test results being reported to public health authorities [18]. We hypothesized that wastewater surveillance data depicted community COVID-19 burden better than clinical test data during the later pandemic period because of decreased clinical testing and under-reporting of home-based COVID-19 test results. We then developed a generalized linear model (GLM) because of non-normal distributions of COVID-19 cases. We fit the GLM with a negative binomial distribution to account for overdispersion of the COVID-19 cases and specified a log-link function. Model fit was assessed using goodness-of-fit measures, including deviance measures and comparison of alternative link functions. These diagnostics indicated that the GLM adequately represented the relationship between predictors and outcome. This incidence density ratio model estimated COVID-19 incidence in the community based on wastewater SARS-CoV-2 concentrations and provided a measure of relative risk, indicating whether the wastewater SARS-CoV-2 concentration at a specific site were associated with a higher or lower incidence of positive clinical cases compared to a reference site. The mathematical formulation of the model is given by:

$$g\left(E\left(Y\right)\right)=\;\beta^{\prime }X$$

where g(.) is a monotone function that relates the expected mean cases to the linear predictors, *Y* is the number of COVID-19 cases, and β is a vector of predictor coefficients corresponding to each site. The expansion of this formula accounted for site-specific effects:

$$g\left(E\left(Y\right)\right)=\;\beta_{A}+\beta_{B}X_{B}+\beta_{C}X_{C}+\beta_{D}X_{D}+\beta_{E}X_{E}+\beta_{F}X_{F}+\beta_{G}X_{G}+\beta_{H}X_{H}+\beta_{I}X_{I}+\beta_{J}X_{J}+\beta X$$

where $\beta_{A}$ is an intercept and represents the effect of site *A*, $\beta_{B}$, …, $\beta_{J}$ represent the effects of each corresponding site, $X_{B}$, …, $X_{J}$ are the binary covariates corresponding to each site, *β* is the effect corresponding to wastewater, and *X* is the wastewater SARS-CoV-2 RNA concentration. We built models that used all the available data (overall), the early pandemic data (pre), and the later pandemic data (post) to predict the number of clinical cases corresponding to each observed wastewater RNA data point. All hypotheses were tested at the standard 5% significance level. Statistical analyses were conducted using SAS version 9.4 (SAS Institute Inc., Cary, NC, USA).

## 3. Results

### 3.1. Descriptive Characteristics

**Figure 1:**
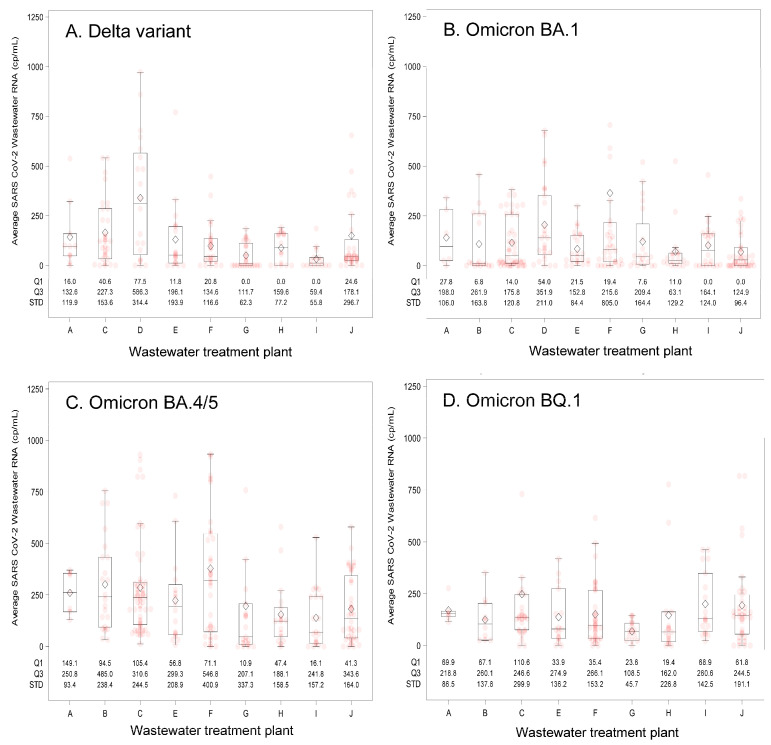
Summary of wastewater SARS-CoV-2 RNA concentrations by wastewater treatment plant (A–J) and predominant circulating SARS-CoV-2 variant. Predominant circulating variants and time periods were: (**A**) Delta variant: 23 May 2021–31 December 2021; (**B**) Omicron BA.1 variant: 1 January 2022–31 May 2022; (**C**) Omicron BA.4/5 variant: 1 June 2022–30 October 2022; and (**D**) Omicron BQ.1 variant: 1 November 2022–17 April 2023. The box and whisker plot lower bound of the box represents quartile 1 (25th percentile) and upper bound quartile 3 (75th percentile) of the data, which together describe the interquartile range. The line bisecting the box represents the 50th percentile (median value). Pink circles indicate individual measured values and diamonds represent the mean value. The whiskers extend 1.5 times the interquartile range, and dots located outside of the end of the whisker are considered outlier values. Abbreviations: cp/mL = copies per milliliter; Q1 = first quartile; Q3 = third quartile; STD = standard deviation.

Characteristics of the 10 participating WWTPs and their associated county attributes appear in Table 1. The WWTP facilities had service populations ranging from 2430 to 35,575 individuals and most (9/10) were in nonmetropolitan counties and had elevated COVID-19 Community Vulnerability Index scores [19]. These WWTPs provided 818 wastewater samples during the surveillance period that began on 23 May 2021, and ended on 17 April 2023. Median wastewater SARS-CoV-2 concentrations varied two-fold across the sites (Table 2) with no clear pattern related to treated wastewater volume, per capita wastewater contribution, or the number of reported COVID-19 cases. The ranges of wastewater SARS-CoV-2 RNA levels observed at each WWTP appear in Figure 1, stratified by the predominate circulating SARS-CoV-2 variant. Measured SARS-CoV-2 wastewater concentrations were on average the highest during the Omicron BA.4/5 period.

### 3.2. Wastewater Correlation with Case-Based Surveillance

**Figure 2:**
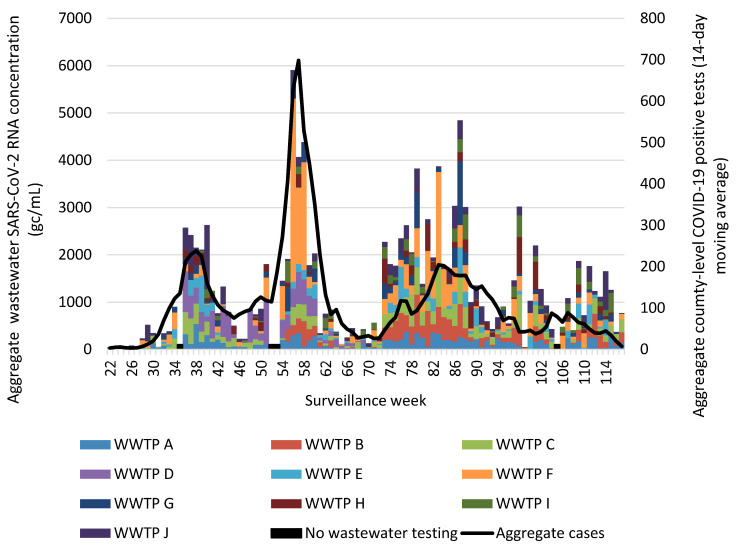
Aggregate weekly wastewater SARS-CoV-2 concentrations from 10 Eastern Kentucky wastewater treatment plants and reported COVID-19 cases from their 9 associated counties, May 2021–April 2023. Aggregated COVID-19 positive tests are presented as a 14-day moving average. Surveillance week 1 corresponds with the first week of January 2021. Black bars represent weeks during which no wastewater testing occurred. Abbreviations: gc/mL = genome copies per milliliter; WWTP = wastewater treatment plant.

**Figure 3:**
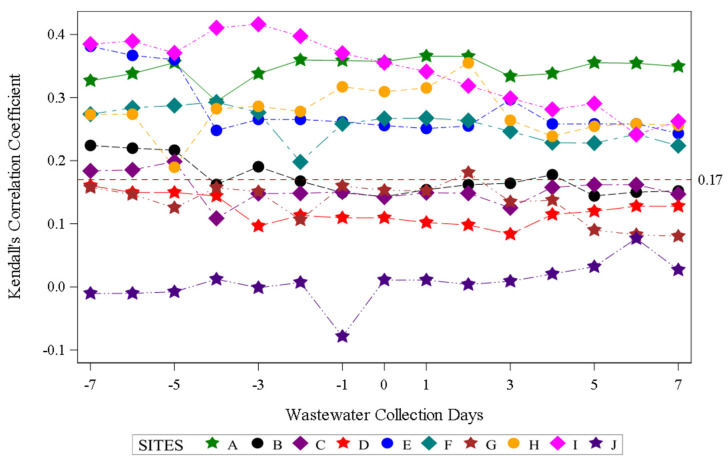
Time-shifted correlation of SARS-CoV-2 wastewater RNA detection and positive COVID-19 clinical test results by site. Negative wastewater collection days indicate the number of days the wastewater data was shifted earlier than the COVID-19 case data, zero indicates no shift, and positive wastewater collection days indicate the number of days the wastewater data was shifted after the COVID-19 case dates. The dotted red line indicates the threshold for statistical significance at α < 0.05.

**Figure 4:**
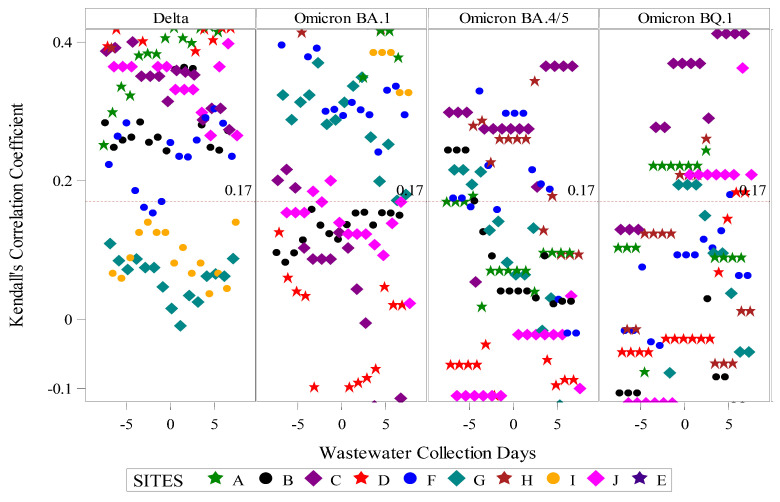
Time-shifted correlation of SARS-CoV-2 wastewater RNA and reported positive COVID-19 clinical tests by site and predominant circulating SARS-CoV-2 variant. Predominant circulating variant time periods were as follows: Delta variant: 23 May 2021–31 December 2021; Omicron BA.1 variant: 1 January 2022–31 May 2022; Omicron BA.4/5 variant: 1 June 2022–30 October 2022; Omicron BQ.1 variant: 1 November 2022–17 April 2023. The dotted red line indicates the threshold for statistical significance at α < 0.05.

Assessment of aggregate wastewater SARS-CoV-2 concentrations and reported COVID-19 cases over time demonstrated a clear visual correlation between the two sources of COVID-19 surveillance data (Figure 2). Site-specific wastewater and clinical case epidemiologic curves showed more variable visual correlations (Figure S1). A time-shifted correlational analysis by WWTP (Figure 3) showed variable correlation between the wastewater signal and COVID-19 case counts by site. Most sites (7/10) showed statistically significant correlations across most of the time shifts. At most locations, correlations were stronger (higher Kendall’s correlation coefficient) when the wastewater data was time-shifted to lead the case data. Correlations were also stronger during the Delta variant-predominant period (Figure 4) than the Omicron variant time periods, and the poorest site level correlations were observed during the Omicron BQ.1 period.

### 3.3. Case Prediction Using Wastewater Data

**Figure 5:**
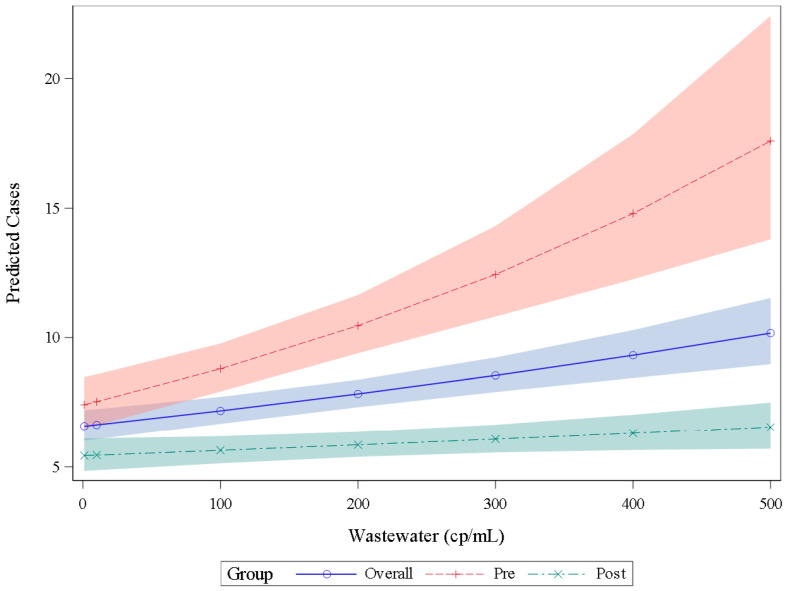
Number of COVID-19 positive clinical tests (cases) predicted by an incidence density generalized linear model using wastewater SARS-CoV-2 RNA concentrations. The blue line (overall) includes all data points regardless of date; predicted cases using the earlier pandemic data set (prior to 30 April 2022) follow the red dashed line (pre) and COVID-19 case predictions using the later pandemic wastewater data (after 30 April 2022) follow the teal dashed line (post). Each prediction uses wastewater data from all locations. The shaded area indicates the prediction interval. Abbreviations: cp/mL = copies per milliliter.

We split the wastewater and clinical case data into earlier (May 2021–April 2022) and later (May 2022–May 2023) data sets. An incidence density model using the earlier dataset (pre) predicted more COVID-19 cases at a given wastewater SARS-CoV-2 concentration compared to the results of the model that used the later data set (post) (Figure 5). For example, at a SARS-CoV-2 wastewater concentration of 200 copies per mL, the model predicted 10 COVID-19 cases using earlier period data and 6 cases when using later period data. Site-specific models varied in their performance in predicting observed COVID-19 cases using wastewater SARS-CoV-2 concentrations (Figure S2); the model using wastewater data aggregated across sites produced case predictions that visually correlated to the reported cases in those counties with an increasing ratio of predicted to reported cases during the latter months (November 2022–March 2023) of our surveillance period (Figure S3).

## 4. Discussion

We implemented WBDS for SARS-CoV-2 in 10 communities in Eastern Kentucky beginning the second year of the COVID-19 pandemic. These sites were among the first rural US communities to have SARS-CoV-2 WBDS. At most sites, WBDS data reflected trends in clinically identified COVID-19 cases.

To implement SARS-CoV-2 wastewater surveillance in rural communities, we addressed many of the barriers state health departments and communities faced in creating WBDS programs. We secured buy-in from local partners by initially connecting through a state-based network of water quality professionals. Our team provided free testing of wastewater samples and critically transported samples from the rural WWTPs to the university-based laboratory. We learned that shipping of wastewater samples was a barrier to site participation in the state-sponsored WBDS program that launched during our second year of surveillance because of the burden it placed on the limited staff at the WWTPs. We solicited and respected each community’s desires about sharing data and created customized dashboards with rapid turnaround of wastewater results. However, the centralized nature of the public health system in Kentucky with substantial oversite at the state level was a barrier to the use of wastewater surveillance data. Local public health officials looked for guidance from the state health department on how to interpret and use the wastewater data, and, at the time of the study, the state had prioritized other COVID-19 response activities and was not providing guidance on the use of WBDS data.

The strength of the correlation between wastewater SARS-CoV-2 RNA concentrations and reported COVID-19 case counts varied between sites. We did not identify specific community or WWTP characteristics associated with poorer correlations. We observed poor correlations at larger and smaller volume WWTPs. There are several potential reasons some sites demonstrated poorer correlations. First, chemicals in the wastewater contributed by industrial effluent may have degraded or inhibited the detection of viral RNA. As described by Bayati et al., common chemicals can destabilize the viral envelope protecting the RNA analyte [20,21]. Second, some participating WWTPs used sewage holding tanks and lift stations, which may have resulted in long transit times and degradation of biomarkers or a delay in the measured SARS-CoV-2 wastewater signal in relation to the deposition of waste from individuals with COVID-19 illness. Third, the geographic mismatch between COVID-19 positive tests, which were reported at the county level, and wastewater SARS-CoV-2 RNA concentrations corresponding to the WWTP service area may have diminished the measured correlations. For some sites, the majority of the county’s residents lived and/or worked within the WWTP service area but for others the WWTP influent represented a smaller fraction of wastewater generated by the county’s residents. Fourth, the proportion of households connected to centralized sewer systems varied across the study counties. Localized COVID-19 outbreaks occurring in unsewered regions of the study counties may have delayed detection in wastewater at WWTPs, as we demonstrated using an agent-based model in one of our study counties [22]. Finally, there were differences between communities in terms of access to COVID-19 testing, which could have resulted in geographically variable COVID-19 ascertainment.

We also observed a diminishing correlation between the wastewater viral signal and reported COVID-19 cases over time. Correlations were most significant during the SARS-CoV-2 Delta wave, with fewer sites demonstrating significant correlations during the Omicron BA.1 and BA.4/5 periods, and the Omicron BQ.1 period had the least number of sites with significant correlations. There are several explanations for this trend. First, COVID-19 case detection and reporting were more robust during the earlier period of the COVID-19 pandemic, with many healthcare settings, schools, and employers routinely testing and reporting results to the State Health Department. Organization-sponsored testing declined later in the pandemic, and individual decision-making also changed with decreased interest in seeking commercial testing. More individuals began to test at home, and the results of home COVID-19 tests were infrequently shared with public health authorities [17,18]. Second, acquired immunity from immunization and prior infection with SARS-CoV-2 increased throughout the pandemic and may have altered the dynamics of SARS-CoV-2 virus shedding in the stool. Additionally, SARS-CoV-2 viral characteristics, such as the duration and intensity of viral shedding, may have changed over time with changes in the predominant circulating variant [23].

We hypothesized that WBDS would become an increasingly more useful and accurate method for monitoring trends in community circulation of SARS-CoV-2 later in the pandemic when public reporting of COVID-19 clinical testing results diminished. In support of our hypothesis, the model predicted more COVID-19 cases than were reported during the later pandemic period at most study sites. The model may have underestimated the number of COVID-19 cases during the later period because variant-specific viral shedding likely differed across the two periods, and increasing levels of acquired immunity may have resulted in lower numbers of SARS-CoV-2 genome copies shed per COVID-19 case [24]. Situating the results of our model among the many other published wastewater epidemiology models is difficult. Systematic reviews of epidemiologic modeling studies have noted that the substantial variability in methods, laboratory and mathematical, make comparisons between studies difficult [25,26]. Furthermore, local context has substantial influence on model parameters and selected outputs (e.g., point prevalence, reproductive number, and hospitalizations), which further limits comparison. Like most published wastewater epidemiology models, we demonstrated that wastewater SARS-CoV-2 signals provide information on clinical cases in the community with variable performance by site and over time.

There are few published evaluations of WBDS for SARS-CoV-2 in rural and smaller communities in North America. A team from Michigan surveilled wastewater from 16 smaller rural communities during the latter half of 2021 and reported moderate to high correlation with COVID-19 clinical cases at 14 of the sites [27]. The shorter surveillance window (6 months) during the Delta wave and the use of a less robust metric of correlation (Spearman’s rank) may explain the stronger correlations reported in the Michigan study as compared to our analysis. However, findings from our study and studies conducted in smaller, rural communities in Michigan [27,28], New England [29], and Canada [7,30] support that WBDS effectively identified SARS-CoV-2 in wastewater and mirrored trends in clinical case reporting.

There are strengths and limitations of this study that may affect the interpretation of the results. The evaluation included 2 years of wastewater surveillance data from 10 independent WWTPs, resulting in a robust wastewater data set that spanned the circulation of several SARS-CoV-2 variants. COVID-19 case counts came from public data, which underestimated the true number of COVID-19 infections because it did not include home test results and individuals who did not get tested for COVID-19. Additionally, COVID-19 case data were reported at the county level, which did not necessarily match WWTP service boundaries, and several WWTPs received sewage from households in adjacent counties. We adjusted our analyses to account for the differences in population size between WWTP users and county populations.

## 5. Conclusions

Wastewater surveillance at small population rural sites provided SARS-CoV-2 surveillance data that visually reflected COVID-19 case trends at most locations. Performance of wastewater surveillance as assessed by the correlations with reported COVID-19 cases was variable and declined over time. Reductions in clinical testing and reporting of test results likely contributed to the decreases in correlation, suggesting that WBDS may have provided more comprehensive surveillance data than case-based surveillance during the latter half of the study period. More research is needed to understand how to best predict COVID-19 incidence from wastewater data, as temporal changes in population immunity, virus variants, and human behavior likely affect model parameters.

## Figures and Tables

**Table 1:** Characteristics of wastewater treatment plants and associated counties participating in SARS-CoV-2 surveillance.

Site	WWTP Characteristics			Wastewater Surveillance		County Characteristics			
	Population Served	Commercial Customers	Annual Volume ^1^	Start Date	End Date	Population ^2^	Sewered (%) ^3^	RUCC ^4^	CCVI ^5^
A	32,575	1491	6942	23 May 2021	17 April 2023	47,754	81	2	0.233
B	4723	761	1507	10 January 2022	17 April 2023	26,438	24	6	0.583
C	15,212	796	4580	5 July 2021	17 April 2023	24,565	60	7	0.133
D	8664	708	1196	22 August 2021	17 April 2022	35,298	33	7	0.342
E	3508	111	556	31 May 2021	17 April 2023	11,490	26	9	0.875
F	2773	18	553	13 June 2021	17 April 2023	22,550	32	7	0.508
G	3679	116	83	5 July 2021	17 April 2023	13,691	15	9	0.650
H	2430	164	363	31 August 2021	8 March 2023	21,229	30	9	0.617
I	8941	439	2763	23 September 2021	9 March 2023	27,929	33	7	0.483
J	5258	426	1234	13 June 2021	29 March 2023	22,550	32	7	0.508

Abbreviations: CCVI = COVID-19 Community Vulnerability Index; RUCC = Rural-Urban Continuity Code; WWTP = wastewater treatment plant. ^1^ In millions of liters. ^2^ County population estimate as of 1 July 2021. Data from US Census Bureau are available at https://www.census.gov/data/tables/time-series/demo/popest/2020s-counties-total.html accessed on 20 February 2026. ^3^ The percentage of households in the county connected to a centralized sewer system. Data from https://kia.ky.gov/WRIS/Pages/Management-Plans.aspx accessed on 20 February 2026. ^4^ Rural-Urban Continuity Codes are used by the US Department of Agriculture to distinguish counties by the size of the population of their metropolitan area and nonmetropolitan counties by their degree of urbanization and proximity to a metropolitan area. Codes range from 1 (counties in metro areas of 1 million population or more) to 9 (urban population of fewer than 5000, not adjacent to a metro area). See https://www.ers.usda.gov/data-products/rural-urban-continuum-codes/documentation# (accessed 28 October 2024) for a detailed description of the methods and nine codes. ^5^ COVID-19 Community Vulnerability Index (CCVI) data are available at https://ky-dph.maps.arcgis.com/apps/MapSeries/index.html?appid=fef62c767a734a1ab2a0140a7477920e, accessed 28 October 2024. The index comprises 34 social, epidemiological, and healthcare systems factors, and the index rank ranges from 0 (least vulnerable) to 1 (most vulnerable).

**Table 2:** Wastewater SARS-CoV-2 RNA concentrations and reported COVID-19 positive tests by surveillance site.

	Wastewater SARS-CoV-2 RNA (cp/mL) ^1^						County-Level COVID-19 Positive Tests (7-Day Moving Average)					
WWTP	*N*	Mean	Median	Std	Min	Max	*N*	Mean	Median	Std	Min	Max
A	88	135.5	114.4	110.7	0	537.6	88	27.4	20.6	30.7	1.7	198.9
B	58	226.1	207.4	202.1	0	806.9	58	4.1	4.1	0.0	4.1	4.1
C	121	199.0	132.1	221.8	0	1360.0	121	11.9	8.9	9.8	0.4	65.9
D	42	270.1	157.7	266.2	0	972.5	42	32.5	20.1	32.1	0.4	109.0
E	70	138.5	65.9	163.6	0	770.3	70	6.6	3.4	7.3	0.3	31.0
F	120	262.0	97.1	486.8	0	3877.0	120	10.0	7.9	8.8	0.4	51.9
G	77	107.8	44.9	196.4	0	1350.2	77	7.4	5.4	8.6	0.1	59.7
H	66	113.5	63.9	156.1	0	776.4	66	11.8	8.8	12.1	0.7	71.6
I	57	120.4	69.9	134.0	0	528.8	57	18.5	11.3	17.8	0	79.0
J	120	151.6	77.1	196.7	0	1360.8	120	10.3	8.1	8.2	0.4	46.3

Abbreviations: cp/mL = copies per milliliter; max = maximum; min = minimum; std = standard deviation; WWTP = wastewater treatment plant. ^1^ Measured wastewater SARS-CoV-2 concentrations presented, e.g., ceiling values not used for these descriptive statistics.

## Data Availability

The original wastewater data presented in the study are available at the NIH RADx Data Hub: https://radxdatahub.nih.gov/study/52. The COVID-19 clinical case data presented in the study are openly available at the New York Times GitHub website: https://github.com/nytimes/covid-19-data.

## Supplementary Materials

The following supporting information can be downloaded at: https://www.mdpi.com/article/10.3390/v18030282/s1, Figure S1: Site-specific wastewater SARS-CoV-2 concentrations and COVID-19 cases over time, Figure S2: Site-specific COVID-19 case predictions based on wastewater SARS-CoV-2 RNA concentrations, Figure S3: COVID-19 case predictions based on aggregate wastewater SARS-CoV-2 RNA concentrations.

## Abbreviations

The following abbreviations are used in this manuscript:

SARS-CoV-2	severe acute respiratory syndrome coronavirus
RNA	ribonucleic acid
WWTP	wastewater treatment plant
CDC	Centers for Disease Control and Prevention
WBDS	wastewater-based disease surveillance
NWSS	National Wastewater Surveillance System
ESP	exclusion-based sample preparation
PMP	paramagnetic particle
Cq	quantification cycle
PCR	polymerase chain reaction
GLM	generalized linear model
RUCC	rural-urban continuity code
CCVI	COVID-19 Community Vulnerability Index
