# Supplementary material for: Wastewater Surveillance for SARS-CoV-2 in Rural Kentucky, 2021–2023
Source: Viruses. 2026 Feb 26;18(3):282. doi: 10.3390/v18030282 (PMC13030766; doi:10.3390/v18030282)
Supplement: Supplementary file 1 [file viruses-18-00282-s001.zip › viruses-4090203-supplementary.pdf]

Supplementary Materials

Wastewater surveillance for SARS-CoV-2 in rural Kentucky, 2021-2023

James W Keck [1,*], Reuben Adatorwovor [2], Ann Noble [3], Savannah Tucker [3], Willam D Strike [4], Soroosh Torabi [3], Mohammad Dehghan Banadaki [3], Blazan Mijatovic [3], Steven K Roggenkamp [5], Donna L McNeill [6], Lindell E Ormsbee [7] and Scott M Berry [3,4]

[1] Department of Family and Community Medicine, University of Kentucky, Lexington, KY 40506, USA; J.K.)
[2] Department of Biostatistics, University of Kentucky, Lexington, KY 40506, USA; (R.A.)
[3] Department of Mechanical and Aerospace Engineering, University of Kentucky, Lexington, KY 40506, USA; (A.N.); (S.T.) (S.T.); (M.D.B.); (B.M.); (S.B.)
[4] Department of Biomedical Engineering, University of Kentucky, Lexington, KY 40506, USA; (D.S.)
[5] Institute for Biomedical Informatics, University of Kentucky, Lexington, KY 40506, USA; s (S.R.)
[6] Kentucky Water Research Institute, University of Kentucky, Lexington, KY 40506, USA; (D.M.)
[7] Department of Civil Engineering, University of Kentucky, Lexington, KY 40506, USA; (L.O.)
* Correspondence: jwkeck@alaska.edu

Figure S1. Site-specific wastewater SARS-CoV-2 concentrations and COVID-19 cases over time
Figure S2. Site-specific COVID-19 case predictions based on wastewater SARS-CoV-2 RNA concentrations
Figure S3. COVID-19 case predictions based on aggregate wastewater SARS-CoV-2 RNA concentrations

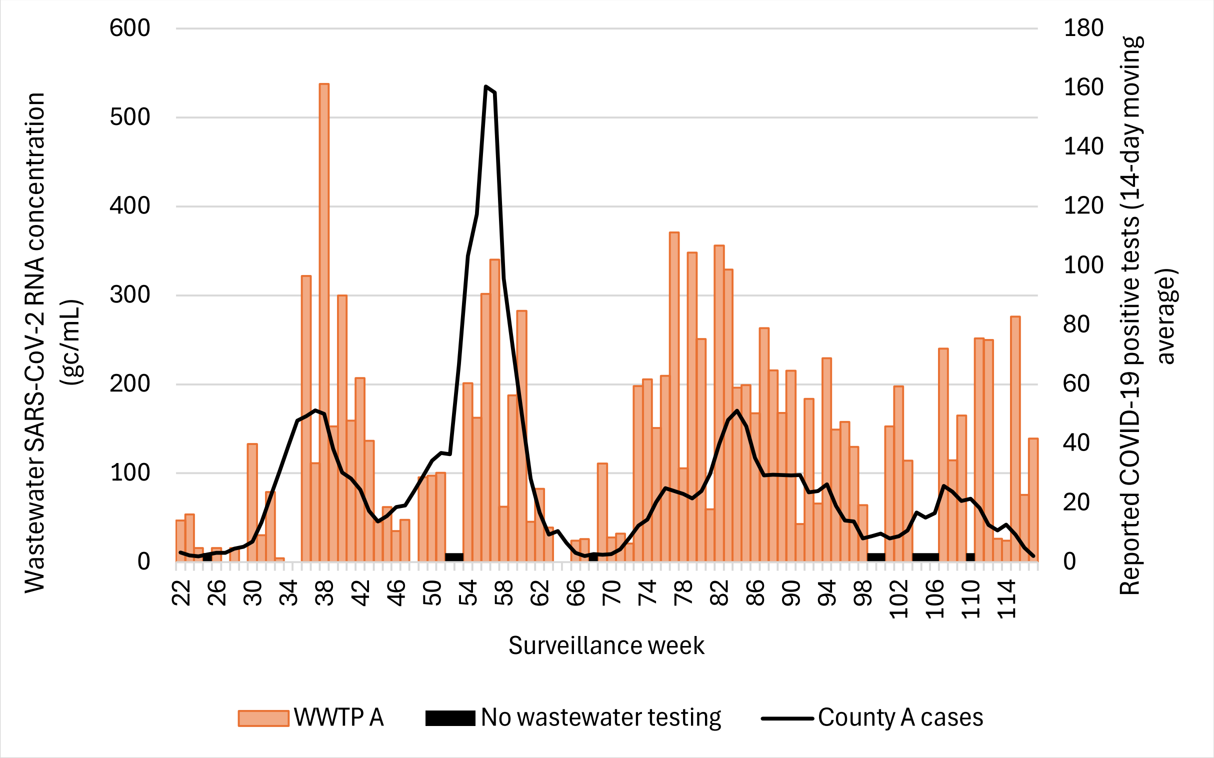
Wastewater SARS-CoV-2 RNA concentration (gc/mL)
600
500
400
300
200
100
0
Reported COVID-19 positive tests (14-day moving average)
180
160
140
120
100
80
60
40
20
0
22 26 30 34 38 42 46 50 54 58 62 66 70 74 78 82 86 90 94 98 102 106 110 114
Surveillance week
WWTP A
No wastewater testing
County A cases

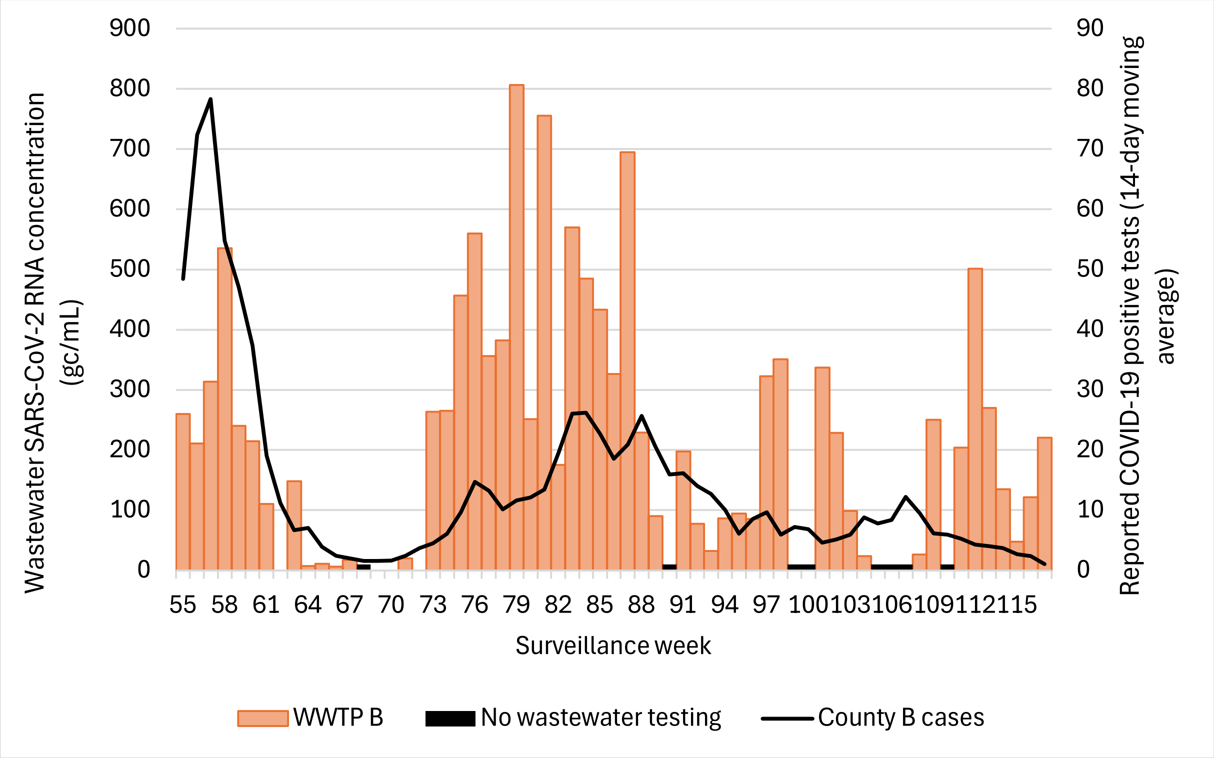
Wastewater SARS-CoV-2 RNA concentration (gc/mL)
900
800
700
600
500
400
300
200
100
0
Reported COVID-19 positive tests (14-day moving average)
90
80
70
60
50
40
30
20
10
0
55 58 61 64 67 70 73 76 79 82 85 88 91 94 97 100 103 106 109 112 115
Surveillance week
WWTP B
No wastewater testing
County B cases

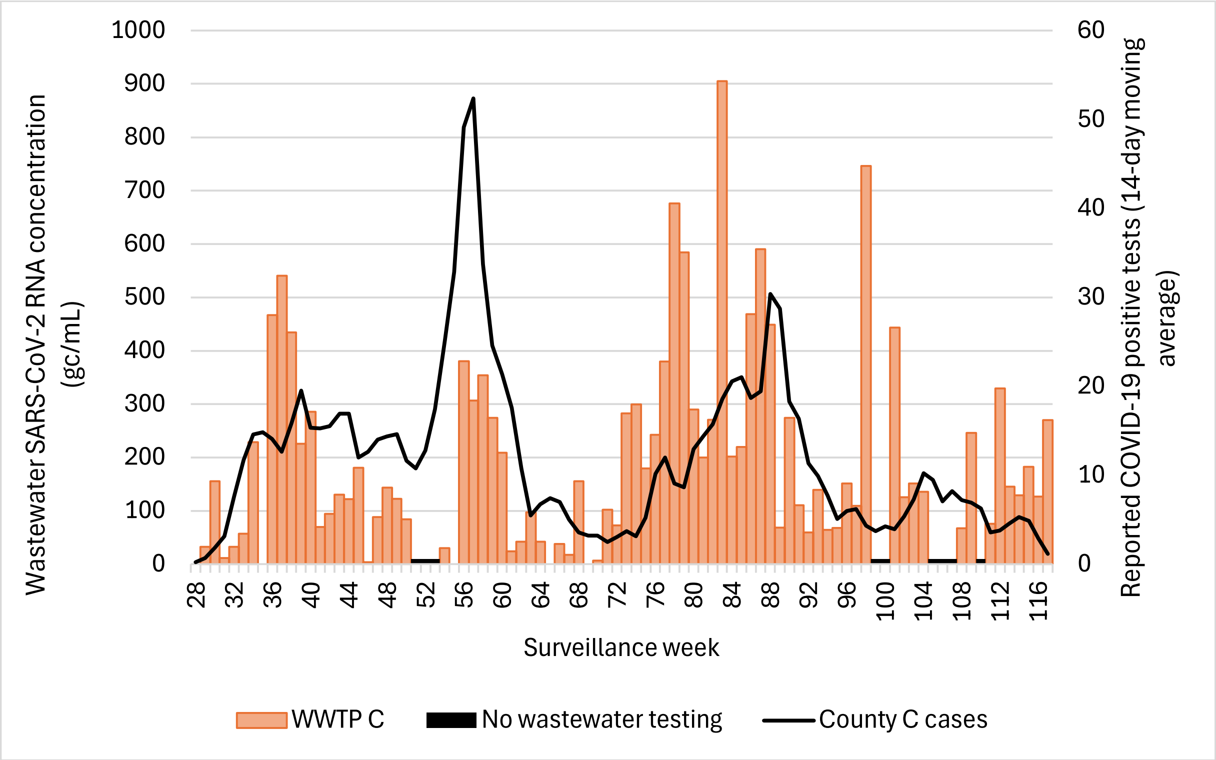

Wastewater SARS-CoV-2 RNA concentration (gc/mL)
Reported COVID-19 positive tests (14-day moving average)
Surveillance week
WWTP C
No wastewater testing
County C cases

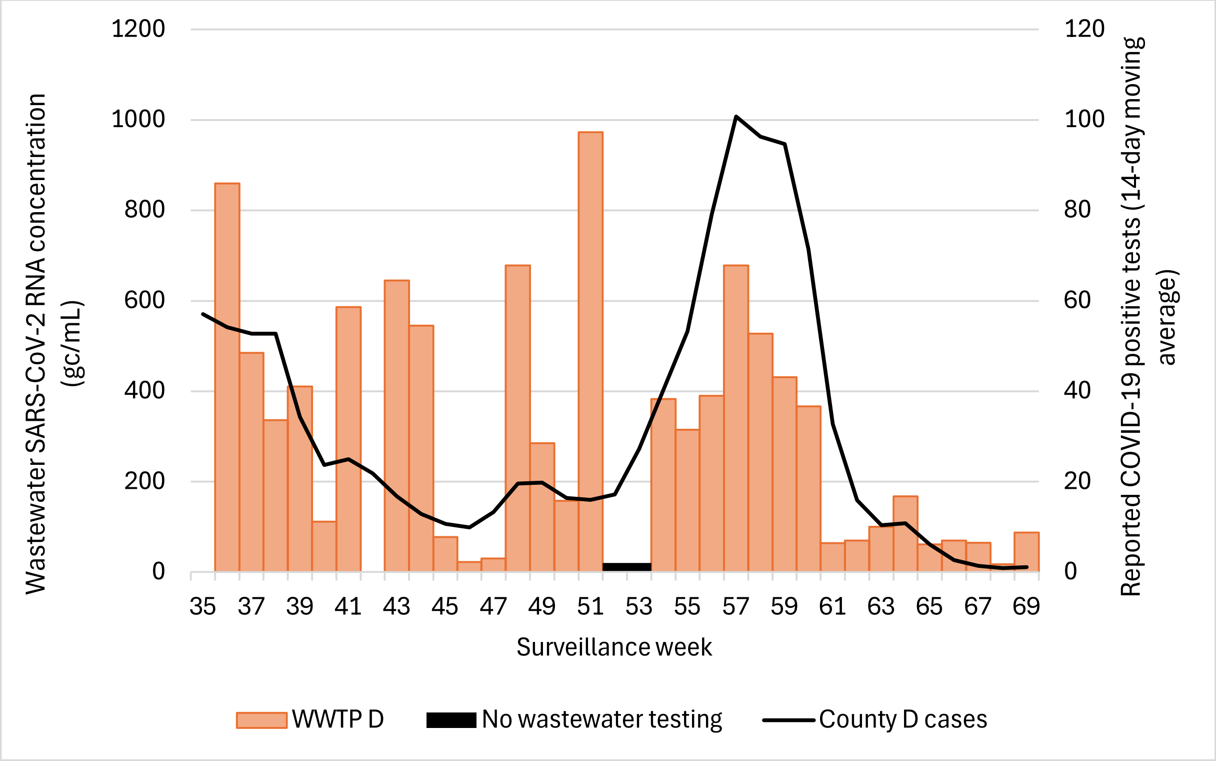

Wastewater SARS-CoV-2 RNA concentration (gc/mL)
Reported COVID-19 positive tests (14-day moving average)
Surveillance week
WWTP D
No wastewater testing
County D cases

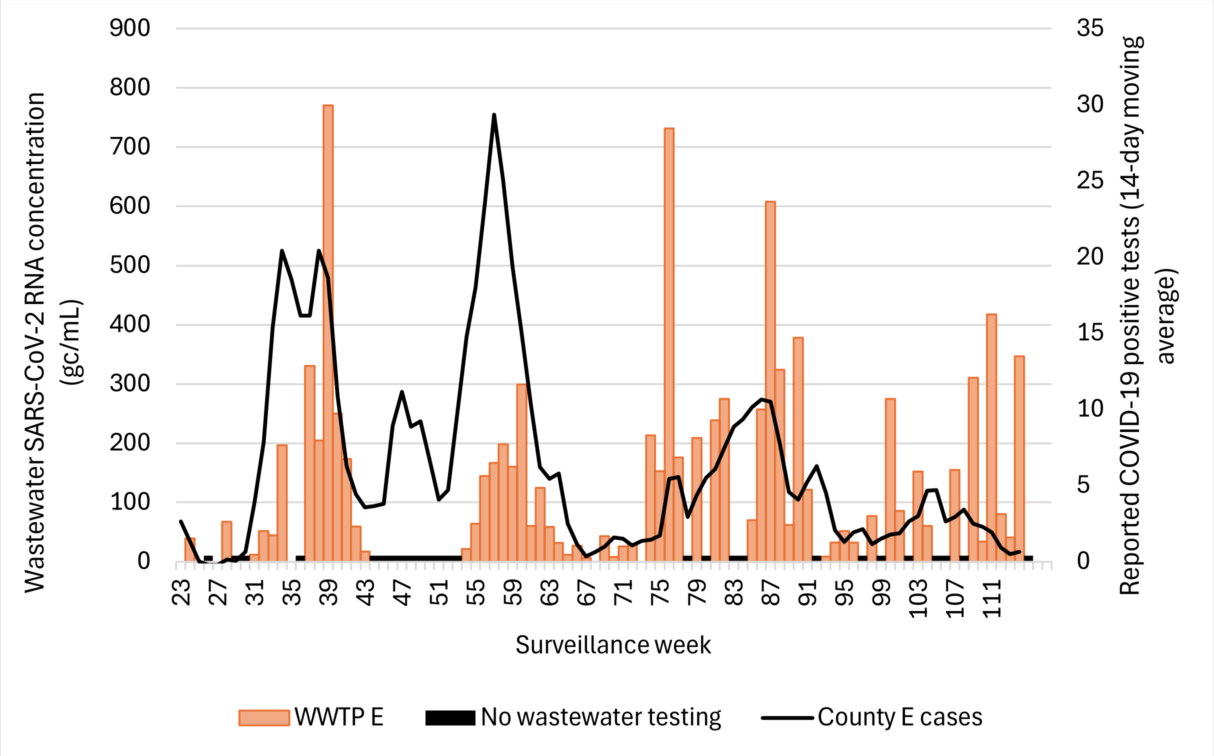
Wastewater SARS-CoV-2 RNA concentration (gc/mL)
Reported COVID-19 positive tests (14-day moving average)
Surveillance week
WWTP E
No wastewater testing
County E cases

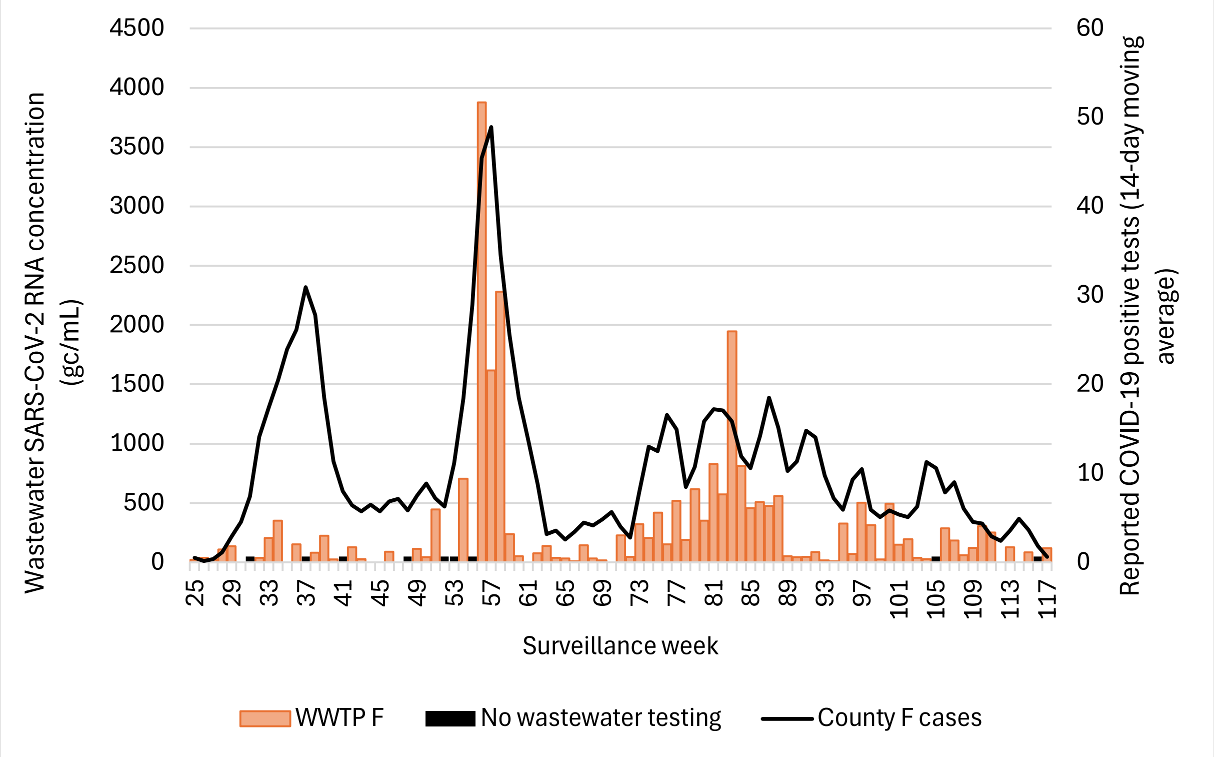
Wastewater SARS-CoV-2 RNA concentration (gc/mL)
Reported COVID-19 positive tests (14-day moving average)
Surveillance week
WWTP F
No wastewater testing
County F cases

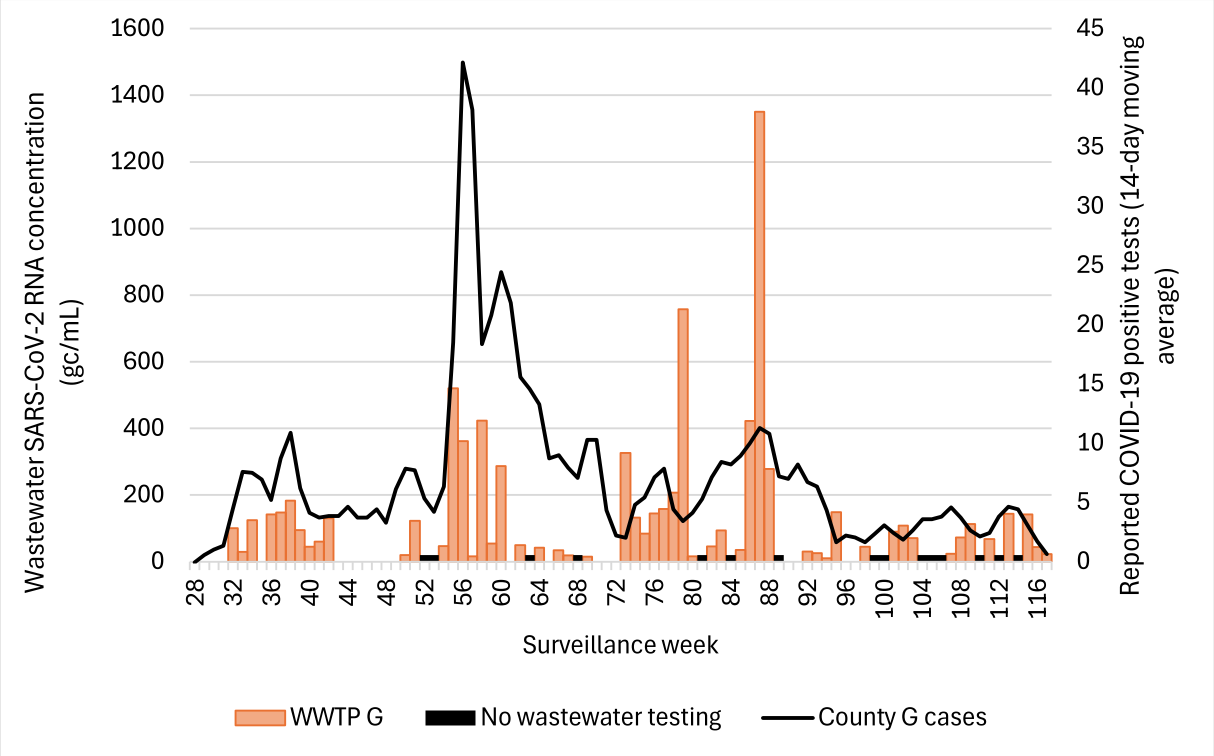
1600
1400
1200
1000
800
600
400
200
0
45
40
35
30
25
20
15
10
5
0
Wastewater SARS-CoV-2 RNA concentration (gc/mL)
Reported COVID-19 positive tests (14-day moving average)
28 32 36 40 44 48 52 56 60 64 68 72 76 80 84 88 92 96 100 104 108 112 116
Surveillance week
WWTP G
No wastewater testing
County G cases

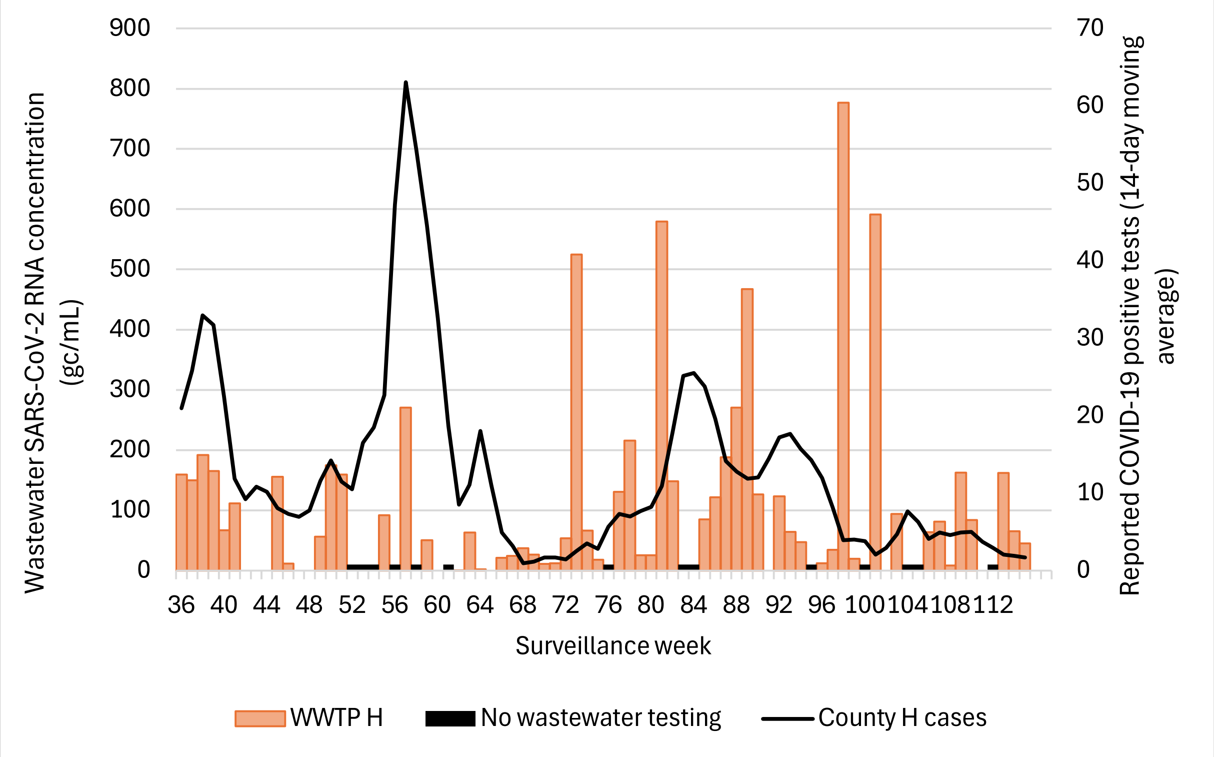
900
800
700
600
500
400
300
200
100
0
70
60
50
40
30
20
10
0
Wastewater SARS-CoV-2 RNA concentration (gc/mL)
Reported COVID-19 positive tests (14-day moving average)
36 40 44 48 52 56 60 64 68 72 76 80 84 88 92 96 100 104 108 112
Surveillance week
WWTP H
No wastewater testing
County H cases

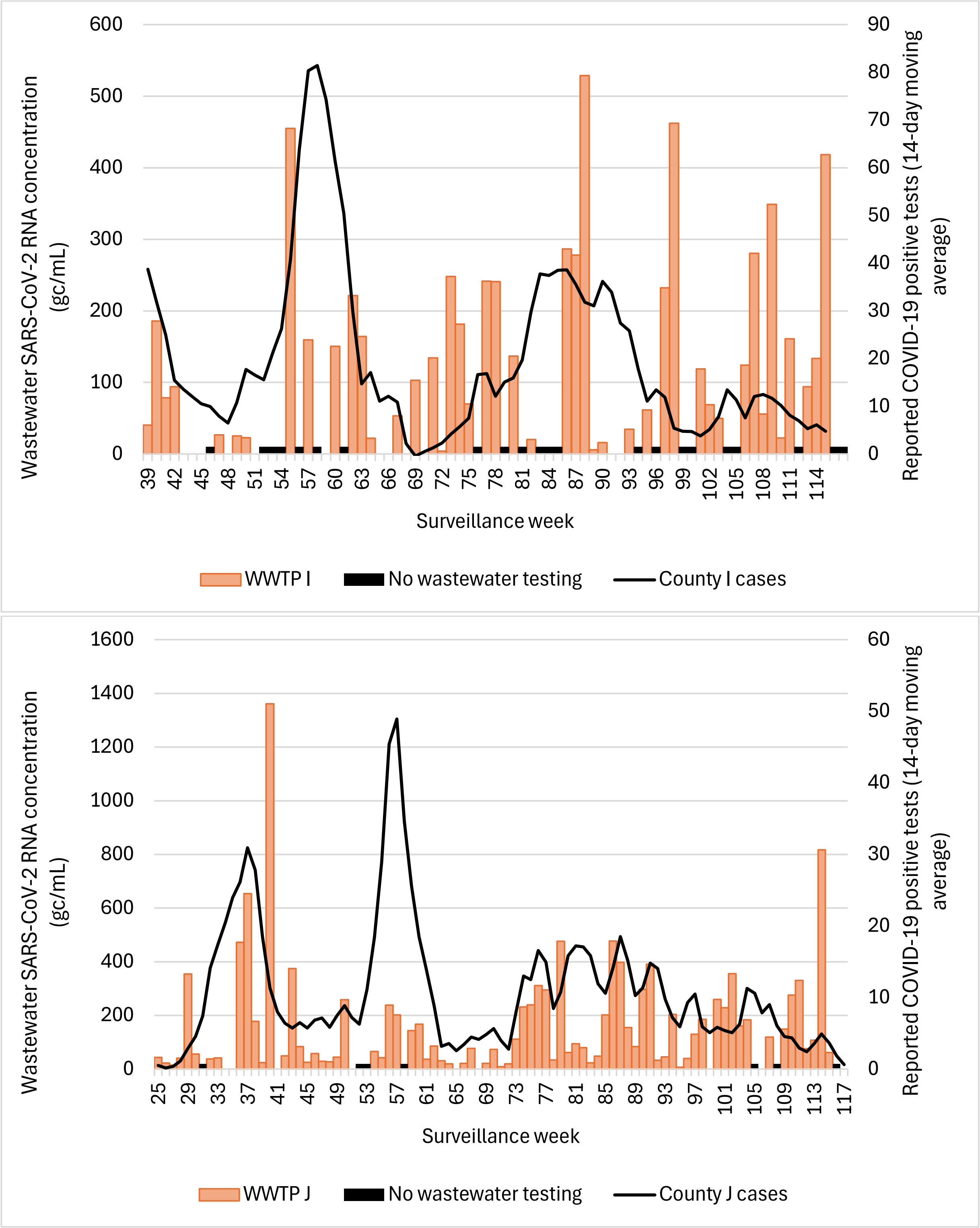

Figure S1. Wastewater SARS-CoV-2 RNA concentrations over time by wastewater treatment plant (**A-J**) shown with county-level reported positive COVID-19 tests. COVID-19 tests presented as a 14-day rolling average. Week 1 is the first week of 2021. Note that y-axis scales vary across sites.

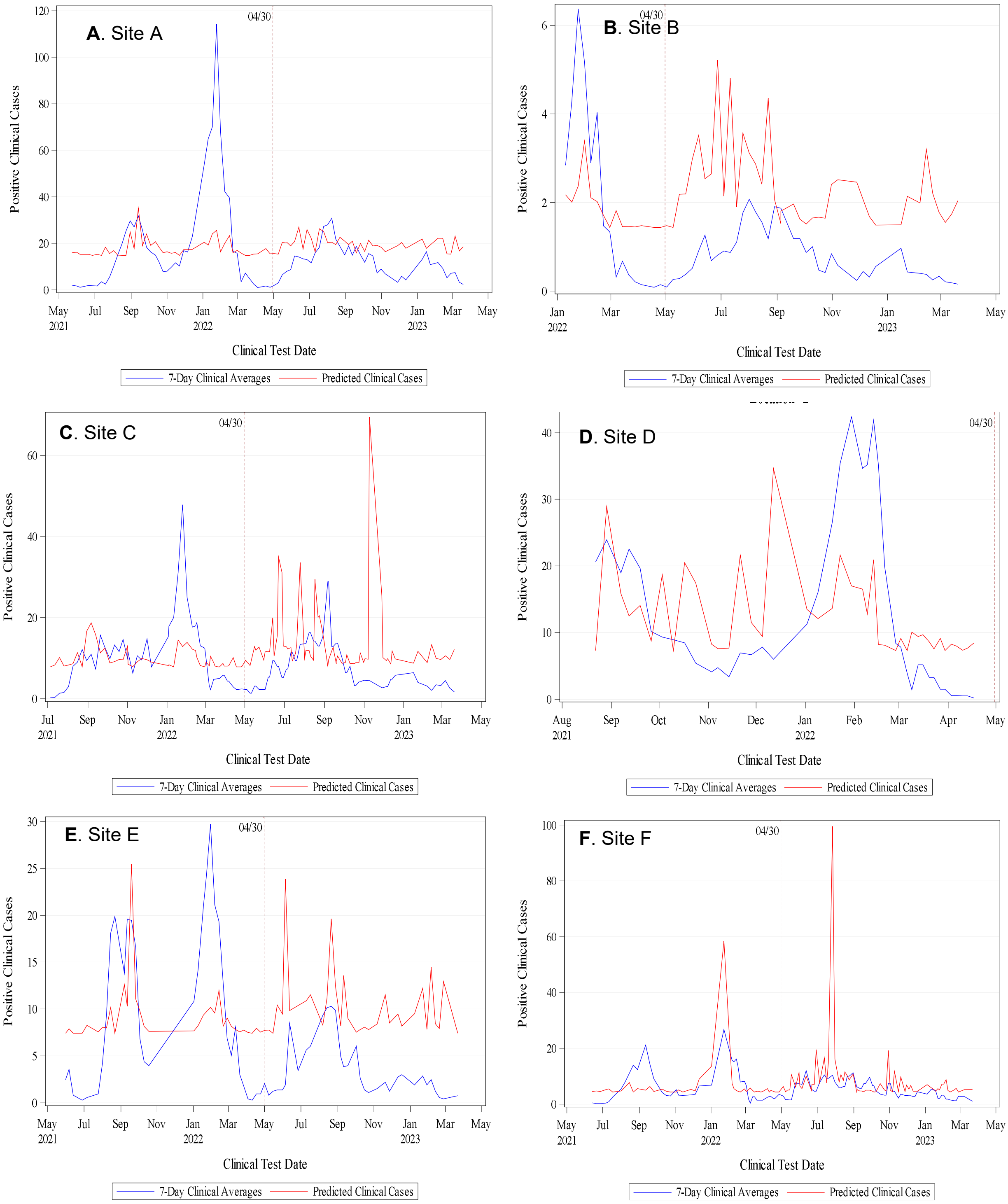

A. Site A
04/30
Positive Clinical Cases
120
100
80
60
40
20
0
May 2021
Jul
Sep
Nov
Jan 2022
Mar
May
Jul
Sep
Nov
Jan 2023
Mar
May
Clinical Test Date
7-Day Clinical Averages
Predicted Clinical Cases
B. Site B
04/30
Positive Clinical Cases
6
4
2
0
Jan 2022
Mar
May
Jul
Sep
Nov
Jan 2023
Mar
May
Clinical Test Date
7-Day Clinical Averages
Predicted Clinical Cases
C. Site C
04/30
Positive Clinical Cases
60
40
20
0
Jul 2021
Sep
Nov
Jan 2022
Mar
May
Jul
Sep
Nov
Jan 2023
Mar
May
Clinical Test Date
7-Day Clinical Averages
Predicted Clinical Cases
D. Site D
04/30
Positive Clinical Cases
40
30
20
10
0
Aug 2021
Sep
Oct
Nov
Dec
Jan 2022
Feb
Mar
Apr
May
Clinical Test Date
7-Day Clinical Averages
Predicted Clinical Cases
E. Site E
04/30
Positive Clinical Cases
30
25
20
15
10
5
0
May 2021
Jul
Sep
Nov
Jan 2022
Mar
May
Jul
Sep
Nov
Jan 2023
Mar
May
Clinical Test Date
7-Day Clinical Averages
Predicted Clinical Cases
F. Site F
04/30
Positive Clinical Cases
100
80
60
40
20
0
May 2021
Jul
Sep
Nov
Jan 2022
Mar
May
Jul
Sep
Nov
Jan 2023
Mar
May
Clinical Test Date
7-Day Clinical Averages
Predicted Clinical Cases

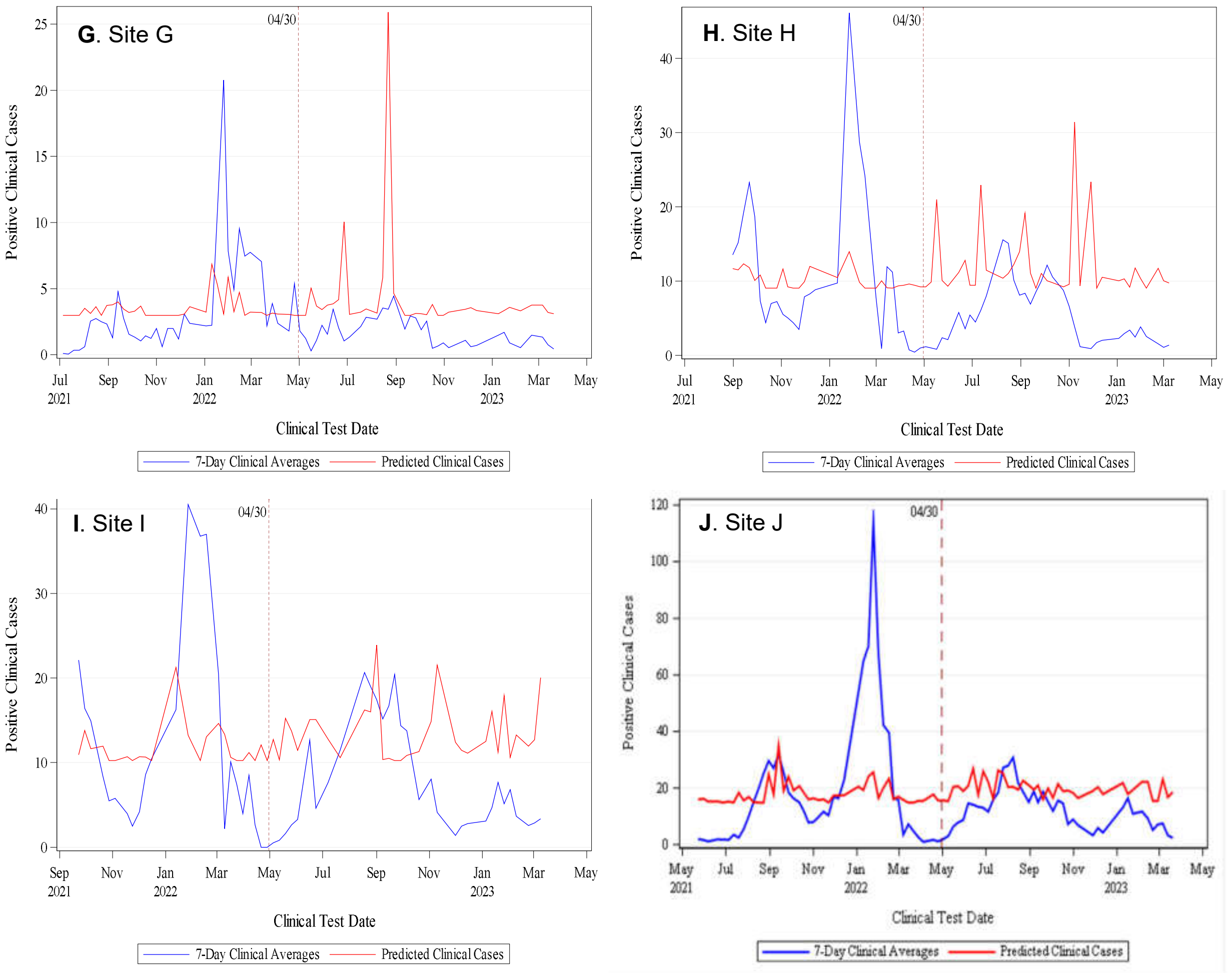

Figure S2. Prediction of COVID-19 cases using wastewater SARS-CoV-2 RNA concentrations by site (panels **A-J**). Each graph shows the reported (blue) and the predicted (red) 7-day average of COVID-19 positive test results over time based on the location's wastewater SARS-CoV-2 RNA concentrations. Wastewater and COVID-19 case data prior to 30 Apr 2022 (indicated by vertical red dashed line) were used to develop the model. One location is shown per graph; date and case count ranges are not uniform.

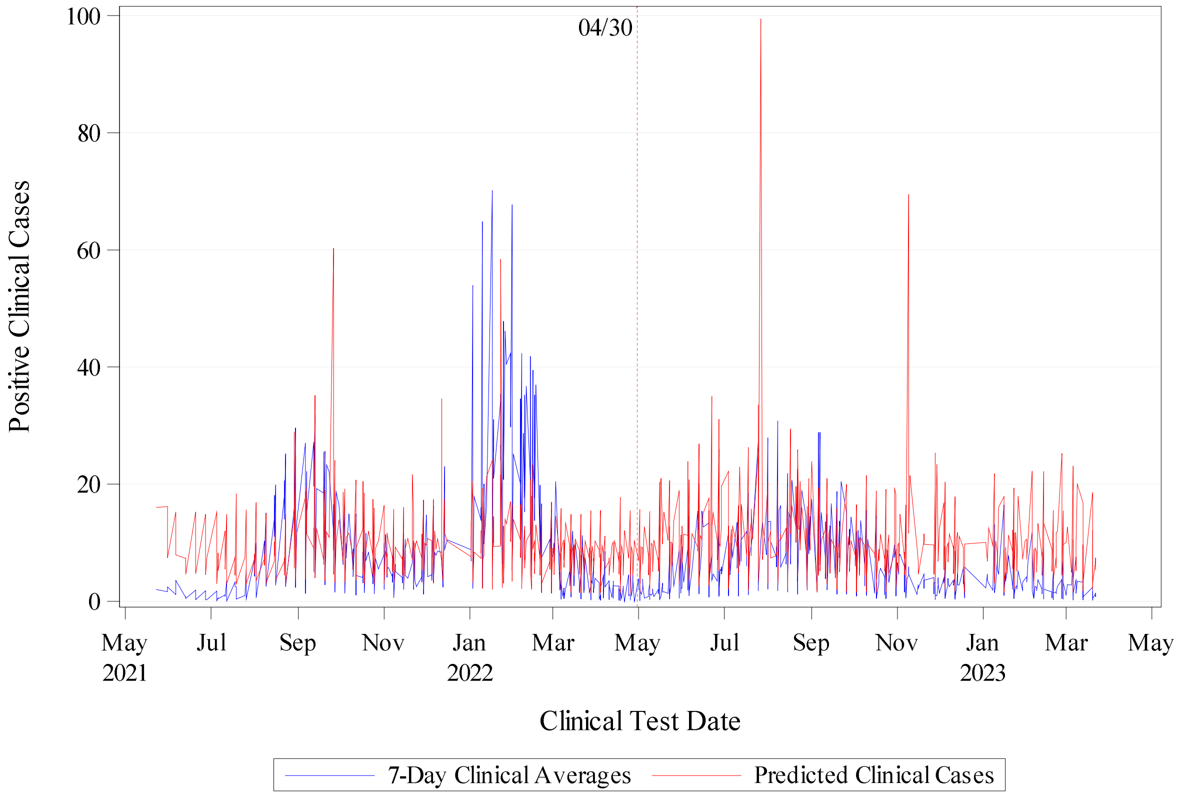

Figure S3. Aggregated prediction of COVID-19 cases using wastewater SARS-CoV-2 RNA concentrations from all sites. The blue line depicts reported positive clinical tests and the red line shows the predicted 7-day average of COVID-19 positive test results based on wastewater SARS-CoV-2 RNA concentrations. Wastewater and COVID-19 case data prior to 30 Apr 2022 (indicated by vertical red dashed line) were used to develop the model. The noisiness reflects periodicity introduced by the timing of clinical case reporting and wastewater testing used to model day-by-day 7-day moving-average predictions without additional smoothing or period aggregation.
